# Citizen Science in Information Systems Research

**DOI:** 10.1007/s12599-020-00663-y

**Published:** 2020-07-29

**Authors:** Christof Weinhardt, Simon Kloker, Oliver Hinz, Wil M. P. van der Aalst

**Affiliations:** 1 Karlsruhe Institute of Technology (KIT), Institute of Information Systems and Marketing (IISM), Kaiserstr. 89, 76131 Karlsruhe, Germany; 2 Faculty of Economics and Business Administration, Goethe University Frankfurt, Theodor-W.-Adorno-Platz 4, 60323 Frankfurt am Main, Germany; 3 RWTH Aachen, Lehrstuhl für Informatik 9, Ahornstr. 55, 52056 Aachen, Germany

## New Ways of Science Communication in Information Systems Research

Science communication is becoming increasingly important, also in information systems (IS) research, as it is increasingly demanded when applying for research funding or academic positions. In 2019, e.g., the German Federal Ministry of Education and Research published a keynote paper highlighting the increasing importance and necessity of appropriate science communication to receive funding (BMBF 2019). Similar requirements have certainly been formulated by many other institutions that provide research funding. According to Lewenstein (2016, p. 1), “Citizen science is one of the most dramatic developments in science communication in the last generation.” Citizen Science, the (large-scale) involvement of citizens in scientific endeavors not only as participants but as co-researchers, is an extreme, but potentially promising, approach to close the gap between scientists and citizens.

In the middle of the 20th century, with the professionalization and institutionalization of science under the supervision of universities and government-run research laboratories, citizens became less involved in science and scientific discoveries (Levy and Germonprez 2017). Science largely excluded the public and became the work of those called scientists. Nowadays, people perceive a big gap between science and everyday life. The post-truth populism is only one of the symptoms of this gap (Smart et al. 2019). We see increased efforts of scientists to communicate their research prolifically, and we see the world consuming it rapidly. However, it seems that it is not always understood correctly (Abraham 2020), which can be observed in some discussions on controversial topics, such as vaccination. This communication gap leaves room for non-scientific messages to spread with the result that some people – or at least some groups – no longer trust scientific findings and scientists at all. Although within the last years this trust has been regained again, there remains a considerable alienation between scientists and citizens, especially among certain groups in the population with low scientific literacy (Gauchat 2011). To increase the scientific literacy of these scientifically disengaged and detached groups, science communication and in particular citizen science can make a huge contribution. Thus, citizen science also contributes to the awareness of the difference between real science and political opinion.

Given the obvious impact of information technology on almost every aspect of society, there are numerous pleas to IS researchers to shoulder their responsibility and address real-world problems (e.g., vom Brocke et al. 2015). Indeed, IS research no longer just focusses on businesses and organizations as it may have done once, but still, our discipline is said to be more concerned with competitive advantages than positive societal impact (Porra and Hirschheim 2007). To communicate these existing efforts of IS researchers to tackle societal issues and make a positive and sustainable impact on society, effective mechanisms of science communication should be used increasingly.

Summing up, what are the objectives of science communication? According to Burns et al. (2003), it is about higher public awareness of science, public understanding of science, scientific literacy among citizens, and finally establishing a scientific culture. In particular, in this editorial, we discuss citizen science in our BISE context and in this context some promising research directions for IS researchers.

## Citizen Science

Lukyanenko et al. (2019a, p. 7) state that despite the importance for the society and the relatedness to our discipline IS “[…] continues to lag behind such disciplines as biology and education in working with citizen science as a context for research.” Disciplines like biology, conservation, and physics are much more active here (demonstrated clearly by Lukyanenko et al. 2019b). Although there are some academic articles in IS literature on citizen science there is still a lack of citizen science projects with clear IS research questions.

A specific definition of citizen science in IS research is provided by Levy and Germonprez (2017, p. 29): “*Citizen science in IS research is a partnership between IS researchers and people in their everyday lives. Citizen science projects in the IS domain involve (a) IS phenomenon of interest to both citizens and scientists, (b) the intervention of citizens in the collection, collaboration, or co*-*creation of scientific endeavors for the purposes of scientific literacy education and a more informed public, and (c) citizens themselves not being the direct subject of scientific inquiry.”* The definition highlights the intended knowledge transfer based on a “learning by doing” research approach and excludes scientific projects that involve the citizens as mere participants of empirical studies and experiments (e.g., using citizen pools). Especially the second constraint is a hard delimitation: Obviously, citizen science should not just make the participants provide their data, but ask them to intentionally collect, deliver and/or use data about research objects – even about the behavior of people (e.g., data about children’s smartphone addiction detected by their parents). Finally, the IS phenomenon needs to be of interest to both researchers and citizens. However, right now we do not find many projects in IS research that meet these criteria. This leads to the question: Do the phenomena we are investigating have the potential to interest and involve the ordinary citizen in a broader scope, or is citizen science within IS research condemned to be the subject of scattered individual projects in niche contexts?

To involve citizens in our research, we first need to have a look at how we can place and generalize our methods and theories in a (sometimes entirely) new context (Lee and Baskerville 2012; Levy and Germonprez 2017). In addition, they need to be comprehensible and applicable to citizens with no noteworthy IS background. Second, we need to attract citizens to our theories, methods, tools, research questions, and fields of interest – basically our knowledge. This will not be possible for all of our work, but let us highlight some methods and fields of research in IS that, inherently, seem fit to this context and which have been addressed (in a related context), among others, in the BISE journal:

- *Participatory Design* (e.g., Qaurooni et al. 2016; Simonofski et al. 2019)
- *Co*-*Creation* (e.g., Haki et al. 2019)
- *User*-*centered Design* (e.g., Grace et al. 2015)
- *User*-*generated Content* (e.g., Tilly et al. 2017)
- *Design Science* (e.g., Mueller et al. 2018)
- *Crowdsourcing/Crowd*-*Reporting* (e.g., Abu-Tayeh et al. 2018; Niemeyer et al. 2018; Schoder et al. 2014)
- *Open Innovation* (e.g., Smart et al. 2019)
- *Gamification* (e.g., Zhou et al. 2017)
- *Ethics,* e.g., regarding *Privacy* (e.g., Krasnova et al. 2012; Peukert and Kloker 2020)

To foster citizen science projects in IS research, we need to increase the interest of our citizens for these kinds of mechanisms. They should not only be interested in using them, but also to research them: invent, test, and evaluate their own mechanisms for, e.g., gamification or crowdfunding – and do so in cooperation with professional researchers. Also, topics like ethics and privacy affect us in current times more than ever before and have a huge potential for citizens to engage in our research, e.g., to understand the use and limitations of the COVID-19 App. The researchers are then responsible for translating the citizen science projects into underlying theories, providing the right infrastructure, training, and tools for observation and measurement (Budde et al. 2017): The *right infrastructure* is important because participation only works on a very low threshold and citizens typically can hardly provide this themselves. Robinson and Imran (2015) declare cost neutrality as the aim, which is even more important in developing countries where the diversity of access devices is high and technological literacy may be rather low (Basole and Karla 2011). The *right training* is important, as information quality is perceived differently by scientists and citizens (Lukyanenko et al. 2016). For scientists, information quality is primarily expressed as consistency and completeness according to a standardized observation protocol. For citizen scientists, quality of information “[…] also includes the extent to which the design of a specific project facilitates citizens’ abilities to spot something interesting, unexpected, or novel” (Lukyanenko et al. 2016, p. 448). Formal training should enable citizen scientists to make exactly this contribution – spot the extraordinary while understanding that ordinary data also has to be recorded seriously. The *right tools for observation and measurement* are important as the lack of experience and a “thrive for the interesting and novel” of citizen scientists remain as a bias in the data (Budde et al. 2017). For this reason, Parsons et al. (2011) advocate that inserting data should be as easy as possible and not compel citizen scientists to make a possibly biased guess. They suggest to let them rather report the observed attributes directly, instead of pressing a classification. Lukyanenko et al. (2019b) showed in a six-month field study and a consecutive laboratory experiment that instance-based user interfaces (reporting of attributes) are better for projects where the focus is on the absolute number of observations and the accuracy of the data, while class-based user interfaces (reporting of classes) are dominant where the focus rather is on precision. In their experiment, citizen scientists reported on plants and animals. For other contexts, these findings may need to be reproduced.

This leads us to a major point in the discussion of citizen science: replication of results. Replication is probably never possible as the research projects are much too dependent on the concrete citizens and surroundings (time, location, …). However, some mechanisms can be used to ensure that each observation is correct – for example, when observations need to be confirmed by at least two independent citizen scientists (Kosmala et al. 2016). Integrity mechanisms of distributed ledger technologies may be of help here (Nofer et al. 2017; Wortner et al. 2019). Further strategies to ensure objectivity, reliability, and validity are, e.g., expert validation or even statistical modeling of systemic error in order to assess the likelihood of false observations (Kosmala et al. 2016). Still, replication of results constitutes a drawback in citizen science that may remain inherent up to a certain degree.

There are also disputable instances of citizen science, at least concerning the definition given at the beginning of this section. One example is the SETI@home project (https://setiathome.berkeley.edu/). At SETI@home, citizens provide the “downtime” of their personal computers to analyze radio telescope data and support the search for extraterrestrial life. While such an approach would result in perfect information quality, it would not be in line with the political and sociological aims of citizen science (a reciprocal shaping of the research project and question, increasing scientific literacy among citizens).

## Research Directions for Citizen Science in Information Systems

Based on the considerations above, the role IS research could play in citizen science is manifold and opens in several research directions:

1. *Technology and Methodology* Participatory design, user-generated content, open innovation, mechanism design, gamification – these are mainly ‘our’ topics and we provide technology and methodology for them. Citizen science projects, in whichever domain they are applied, can profit from our research – and we should name these contributions in our research papers as well, if appropriate. We can also keep the needs of citizen science in our mind while researching one of the relevant methodologies or even do this in or for the context of citizen science.
2. *Information Quality* Problems with information quality is currently one of the major drawbacks of citizen science (Wiggins and Crowston 2015). The challenges of creating a reliable dataset collected by heterogeneous contributors in heterogeneous environments with heterogeneous devices are yet not sufficiently addressed. Inputs, for example, can be generated by photography, geolocation, website tagging, classification tasks, measurement of environmental variables, …. According to Wiggins and Crowston (2015), still more than 70% of all citizen science projects need the support of expert reviews to ensure high quality. Less than 20% can automatically filter bad reports. For this and other reasons, Lukyanenko et al. (2019b) regard information quality issues in citizen science as a unique opportunity for IS researchers to contribute to research in a multitude of other disciplines.
3. *New projects in the BISE community* Finally, our discipline should consider citizen science as a real opportunity to engage citizens in and attract them to our research. So far, there are very few examples, like WYRED, which was conducted in Belgium to identify gender stereotypes on the internet (García-Holgado et al. 2020), that are somehow related to our field. However, many societal challenges are related to information systems today and may open opportunities for citizen science projects. To give just some examples: addiction to information systems, flexibility in energy consumption, sustainability, and crisis management (Huber et al. 2020; Irwin 1995; Kloker 2020; McCormick 2012).

Facing a fast-growing degree of digitalization everywhere, we would like to invite you to make up your minds and think about how to frame research questions in a way that is relevant for society and has impact on it. Starting citizen science projects may be one promising option. Up to now, almost all citizen science projects have mainly been borne by sub-communities such as hobby biologists, hobby geologists, etc. Thus, we are very curious about which groups will be interested in and engage in ‘our’ research topics and citizen science projects. Instead of making the impression that we do not care and pretend to know too little, we should use our full range of capacities to effectively keep records of what is happening around us within these projects. Technology evolves very fast, and we believe that it is the turn of IS researchers to establish scientific literacy and culture among the citizens to face these developments. We’d like to encourage you – and are happy to receive your manuscripts.
